# Supplementary material for: The Effect of C60 and Pentacene Adsorbates on the Electrical Properties of CVD Graphene on SiO2
Source: Nanomaterials (Basel). 2023 Mar 22;13(6):1134. doi: 10.3390/nano13061134 (PMC10052095; doi:10.3390/nano13061134)
Supplement: Supplementary file 1 [file nanomaterials-13-01134-s001.zip › nanomaterials-2283124-supplementary.pdf]

nanomaterials

Supplementary Materials

The Effect of C60 and Pentacene Adsorbates on the Electrical Properties of CVD Graphene on SiO_2

Jacopo Oswald [1,2,*], Davide Beretta [1,*], Michael Stiefel [1,†], Roman Furrer [1], Dominique Vuillaume [3] and Michel Calame [1,2,4,*]

[1] Empa, Swiss Federal Laboratories for Materials Science and Technology, Transport at Nanoscale Interfaces Laboratory, Überlandstrasse 129, CH-8600 Dübendorf, Switzerland
[2] Swiss Nanoscience Institute, University of Basel, Klingelbergstrasse 82, CH-4056 Basel, Switzerland
[3] Centre National de la Recherche Scientifique, Institute for Electronic, Microelectronic and Nanotechnology (IEMN), 59652 Villeneuve d'Ascq, France
[4] Department of Physics, University of Basel, Klingelbergstrasse 82, CH-4056 Basel, Switzerland
* Correspondence: jacopo.oswald@empa.ch (J.O.); davide.beretta@empa.ch (D.B.); michel.calame@empa.ch (M.C.)
† Current address: IBM Zürich Research Laboratory, Säumerstrasse 4, CH-8803 Rüschlikon, Switzerland.

Atomic Force Microscopy (AFM)

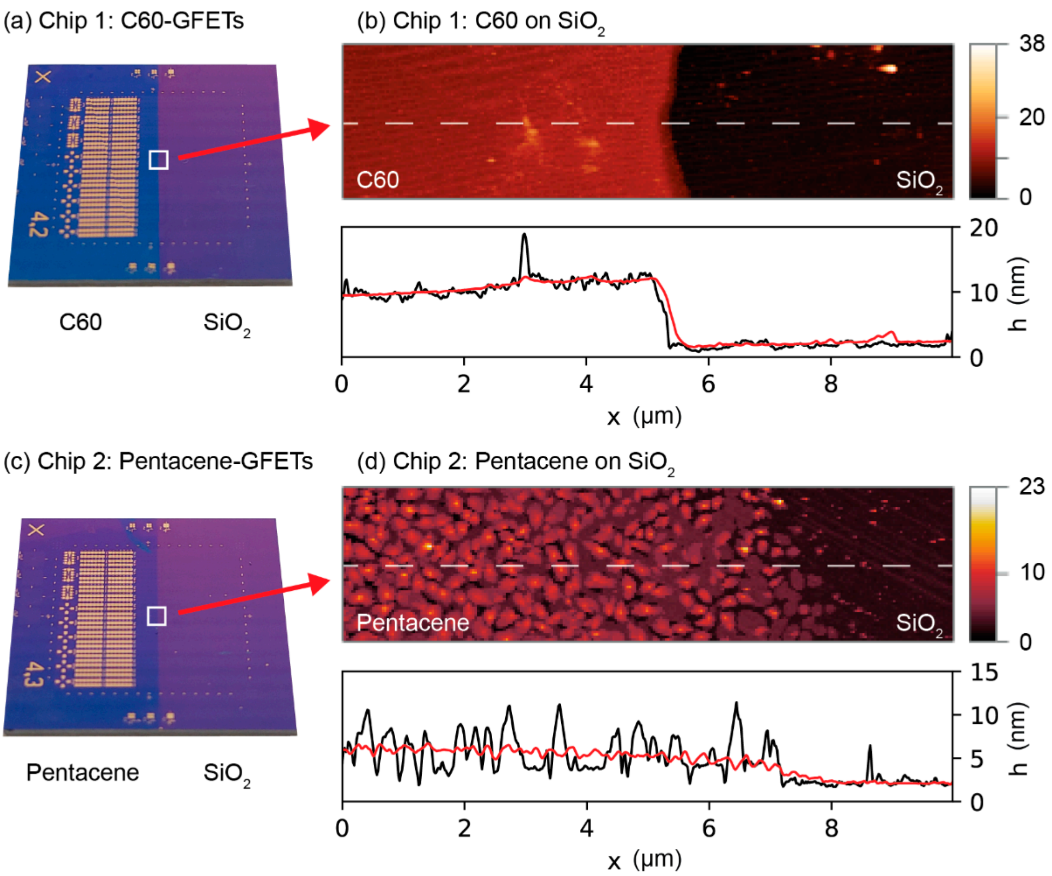

Figure S1. (**a**) Photograph of Chip 1 including the C60-GFETs. The white square show the region where the thickness of the C60 film was measured. (**b**) AFM height image and profile taken on Chip 1 with the set of C60-GFETs. The right part, which was covered during the thermal evaporation of C60, shows the bare SiO_2 substrate. The AFM profile shows that the C60 film on SiO_2 is uniform and the measured average thickness is ca. 10 nm. The black line is the AFM profile taken along the white dashed line on the AFM image. The red line is the mean profile value of the whole image. (**c**) Photograph of Chip 2 including the Pentacene-GFETs. (**d**) AFM height image and profile taken on the chip with the set of Pentacene-GFETs. The right part, which was covered during the thermal evaporation of Pentacene, shows the bare SiO_2 substrate. The Pentacene film is covering large part of the substrate and shows the typical thin film phase structure of Pentacene with an average thickness of ca. 5 nm. The red line is the mean profile value of the whole image.

Raman Spectroscopy

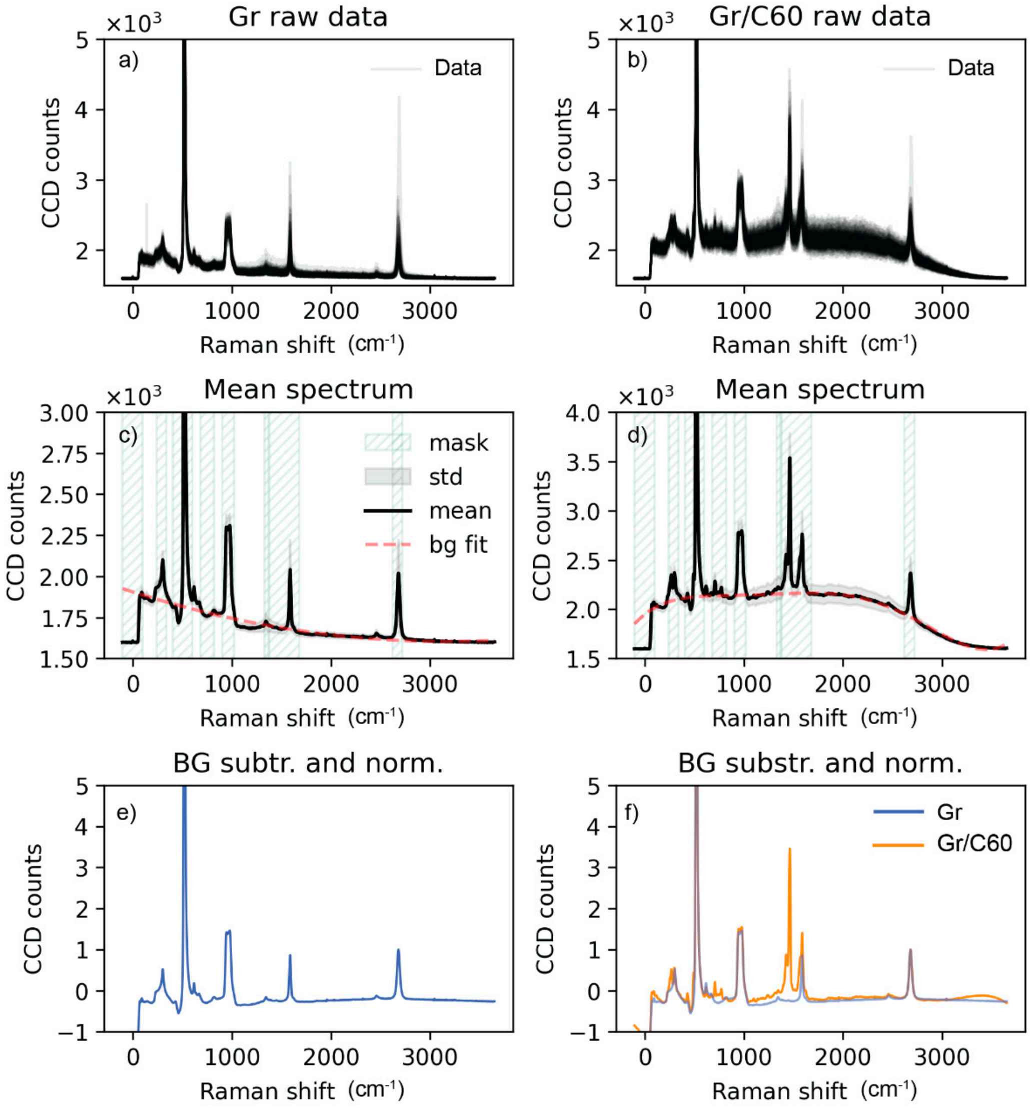

Figure S2. Raman spectrum of the Gr/C60 hybrid heterostructures. (**a**) Raman spectra of the 150 pristine CVD Gr channels. (**b**) Raman spectra of the 150 Gr/C60 channels. (**c**) Average Raman spectrum of the 150 measurements of the pristine CVD Gr in (a). The red dashed line is the polynomial fit (degree 2) of the background. The green hatched areas (mask) are not considered in the polynomial fit. (**d**) Average Raman spectrum of the 150 measurements of the Gr/C60 heterostructures in (b). The red dashed line is the polynomial fit (degree 5) of the background. The green hatched areas (mask) are not considered in the polynomial fit. (**e**) Pristine CVD graphene Raman spectrum after background removal and normalization to the 2D peak of graphene. (**f**) Raman spectrum of the Gr/C60 heterostructure after background removal and normalization to the 2D peak of graphene.

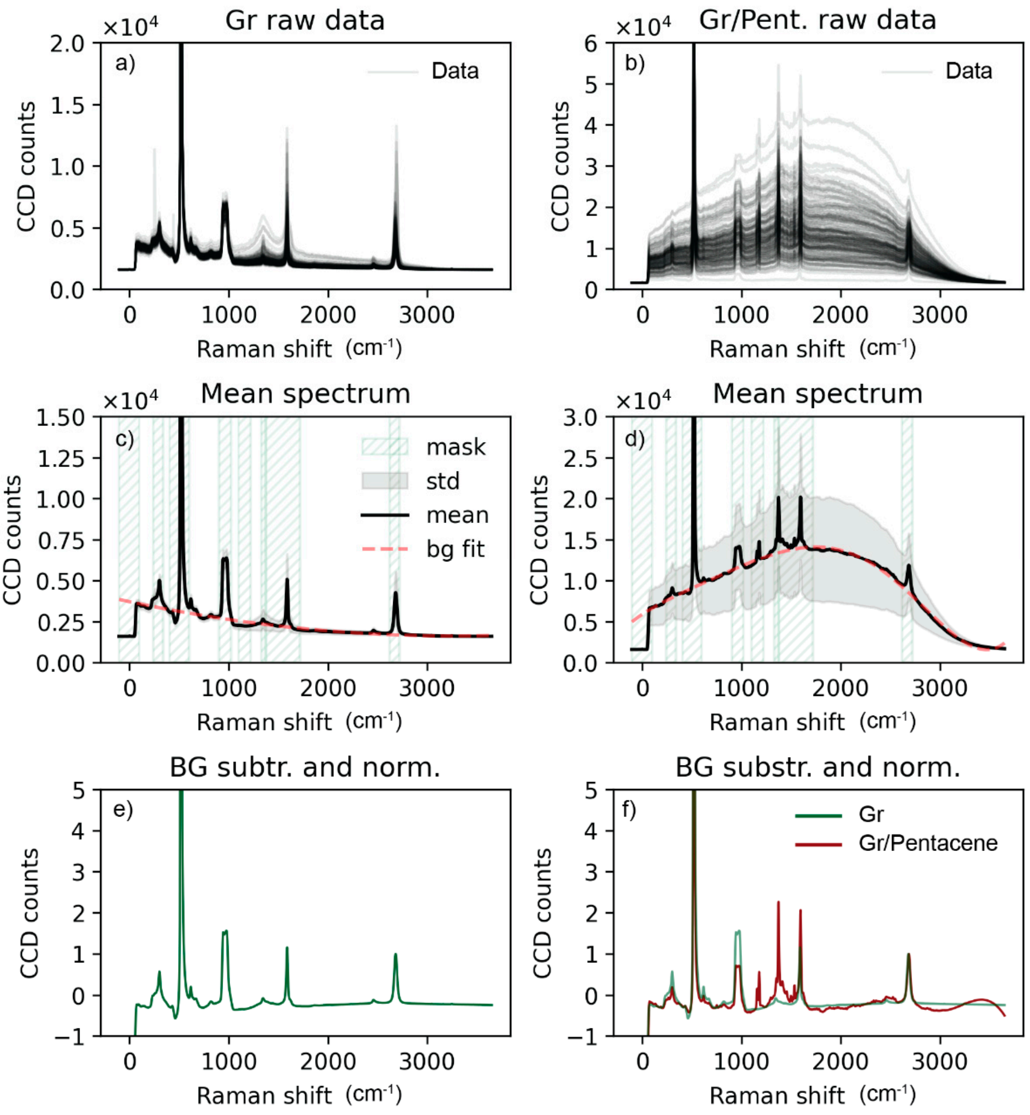

Figure S3. Raman spectrum of the Gr/Pentacene heterostructures. (**a**) Raman spectra of the 150 pristine CVD Gr channels. (**b**) Raman spectra of the 150 Gr/Pentacene channels. (**c**) Average Raman spectrum of the 150 measurements of the pristine CVD Gr in (a). The red dashed line is the polynomial fit (degree 2) of the background. The green hatched areas (mask) are not considered in the polynomial fit. (**d**) Average Raman spectrum of the 150 measurements of the Gr/Pentacene heterostructure in (b). The red dashed line is the polynomial fit (degree 5) of the background. The green hatched areas (mask) are not considered in the polynomial fit. (**e**) Pristine CVD graphene Raman spectrum after background removal and normalization to the 2D peak of graphene. (**f**) Raman spectrum of the Gr/Pentacene heterostructure after background removal and normalization to the 2D peak of graphene.

Electrical Characterization

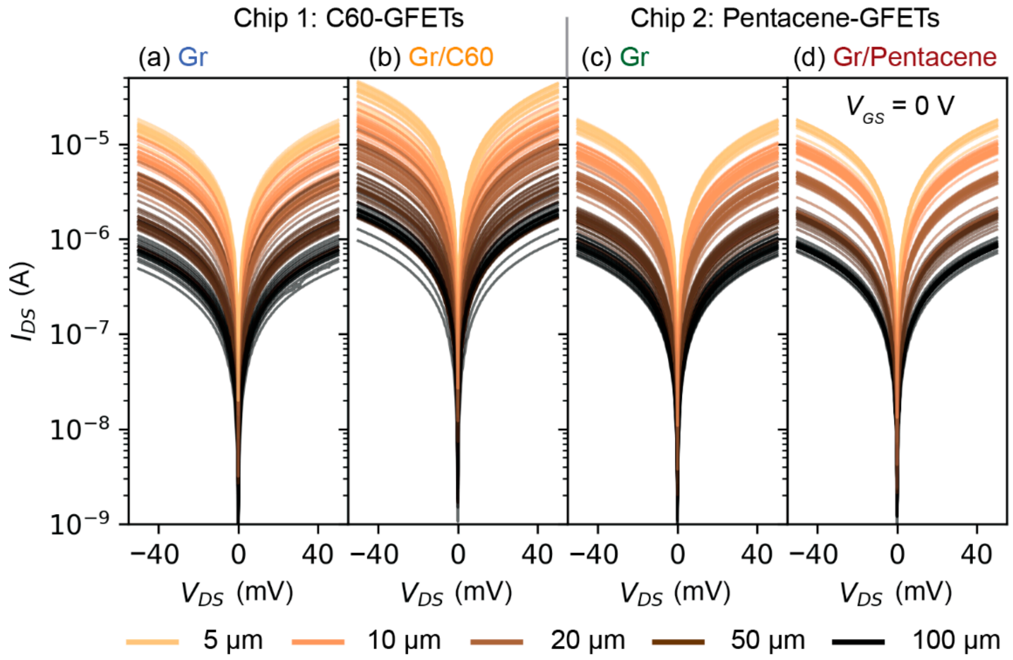

Figure S4. Output characteristic (I_{DS} vs. V_{DS}) of the GFETs measured at zero gate-to-source voltage (V_{GS} = 0 V). (**a**) Output characteristics of pristine CVD graphene FETs before C60 deposition (Chip 1, 101 devices, all *L* included). (**b**) Output characteristics of the C60-GFETs (Chip 1, 101 devices, all *L* included). (**c**) Output characteristics of pristine CVD graphene before Pentacene deposition (Chip 2, 119 devices, all L included). (**d**) Output characteristics of the Pentacene-GFETs (Chip 2, 98 devices, all *L* included).

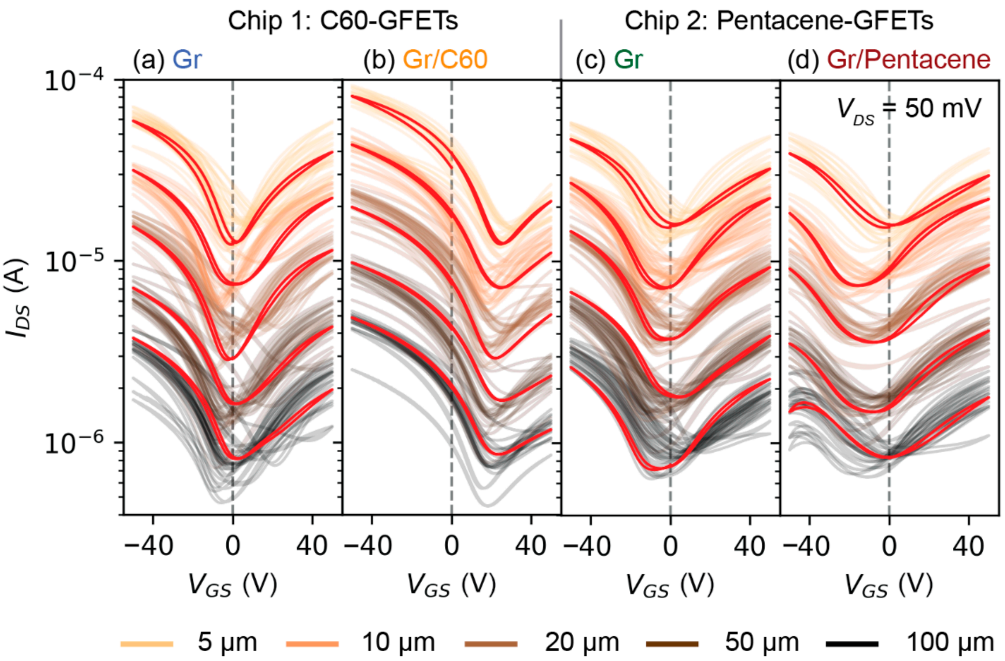

Figure S5. Transfer characteristics (I_{DS} vs. V_{GS}) of the GFETs, the drain-to-source voltage is kept constant at V_{DS} = 50 mV for all measurements. The graphs show a representative forward/backward sweep for each channel length highlighted in red. The backward and forward sweep are virtually identically and only the backward continuous sweep is considered in this work. (**a**) Transfer characteristics of pristine CVD graphene FETs before C60 deposition (Chip 1, 101 devices, all *L* included). (**b**) Transfer characteristics of the C60-GFETs (Chip 1, 101 devices, all *L* included). (**c**) Transfer characteristics of pristine CVD graphene before Pentacene deposition (Chip 2, 119 devices, all *L* included). (**d**) Transfer characteristics of the Pentacene-GFETs (Chip 2, 98 devices, all *L* included).

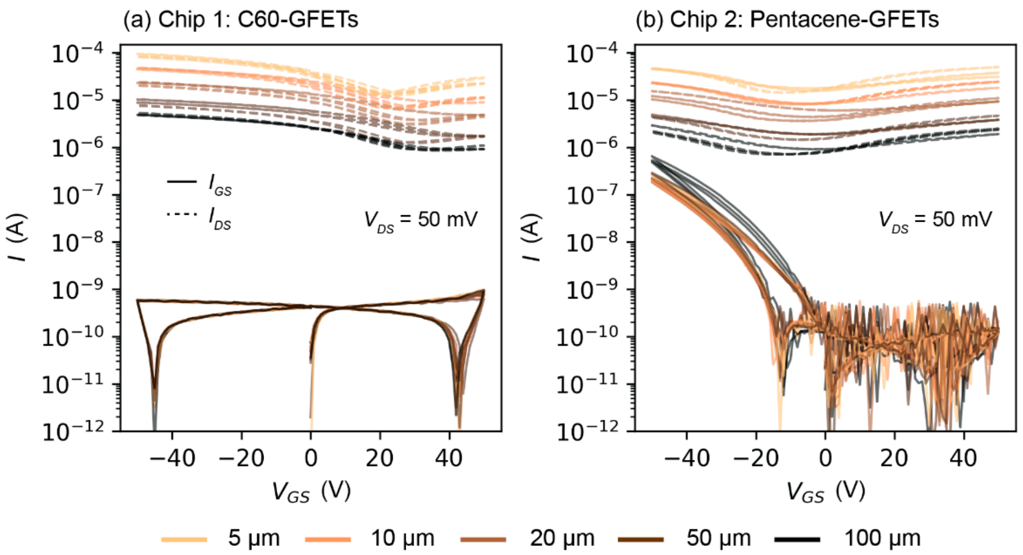

Figure S6. GFETs I_{GS} (gate-to-source current) vs. I_{DS} of 3 representative devices per channel length *L*. (**a**) C60-GFETs. $I_{GS} << I_{DS}$ for the whole gate-to-source voltage range (−50 V to +50 V). (**b**) Pentacene-GFETs. $I_{GS} << I_{DS}$ for positive V_{GS}. The I_{GS} increases for negative gate voltages and the ratio $I_{DS}/I_{GS} \sim 10$ for $V_{GS} < -40\,V$ for the longer graphene channels (*L* = 100 µm). $I_{DS}/I_{GS} > 100$ for the shorter channels in the whole V_{GS} range (−50 V to +50 V). The increasing I_{GS} for negative V_{GS} in Pentacene-GFETs is possibly ascribed to the gating of the full film of Pentacene (*p*-type OSC), which becomes more conductive at negative gate voltages. Since I_{GS} is not limited, the transfer characteristics of the GFETs and the conclusions of this work are not affected.

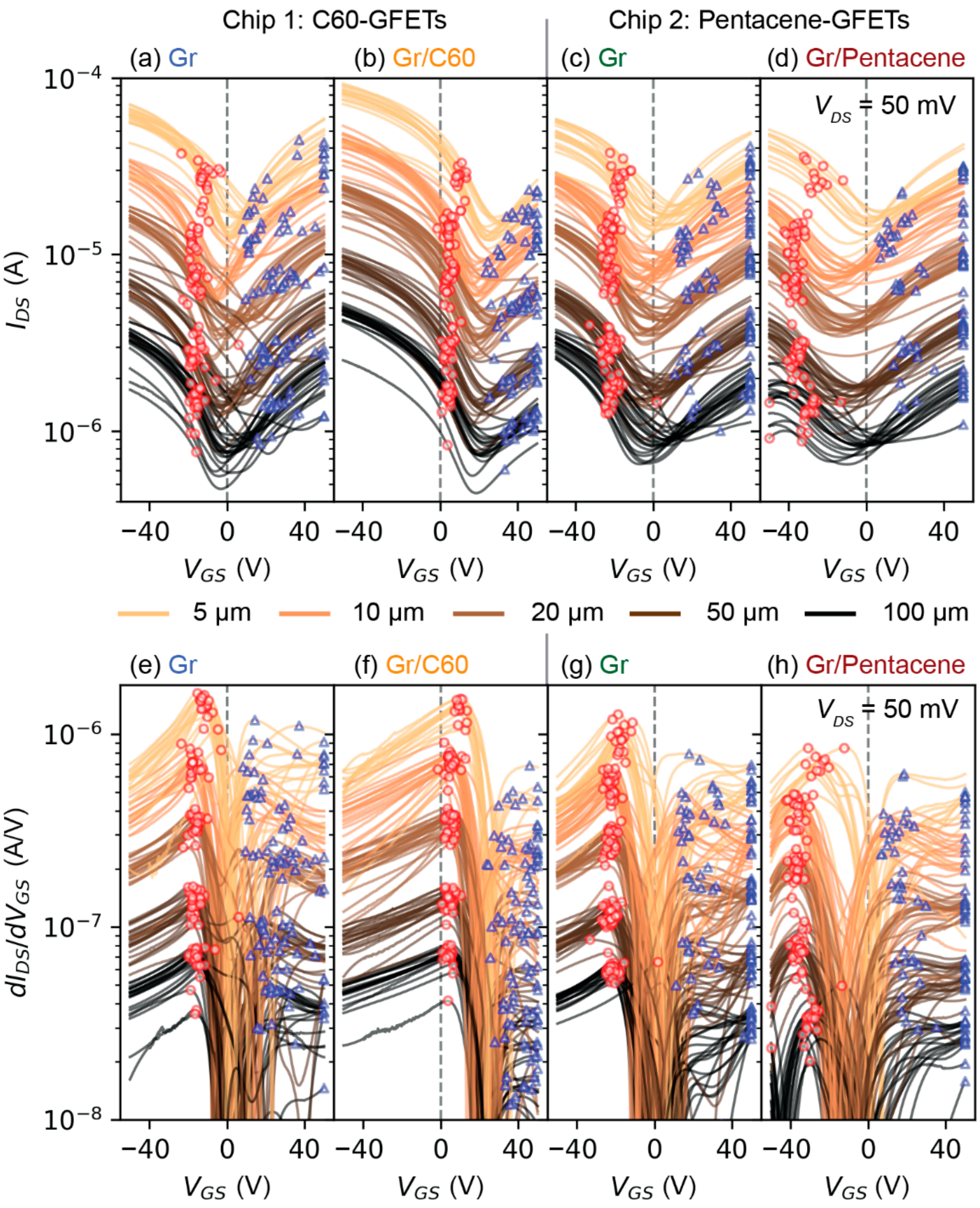

Figure S7. (**a**)–(**d**) Transfer characteristics (I_{DS} vs. V_{GS}) of the GFETs before and after the deposition of the C60 and Pentacene molecules. (**e**)–(**h**) Numerical derivative of the transfer characteristics (dI_{DS}/dV_{GS} vs. V_{GS}) of the GFETs before and after the deposition of the C60 and Pentacene molecules. The red circles show the maximum of the derivative for $V_{GS} < V_{GS}^{Dirac}$ that is used to calculate the field effect hole mobility using equation 2. The blue triangles show the maximum of the derivative for $V_{GS} > V_{GS}^{Dirac}$ that is used to calculate the field effect electron mobility using equation 2.

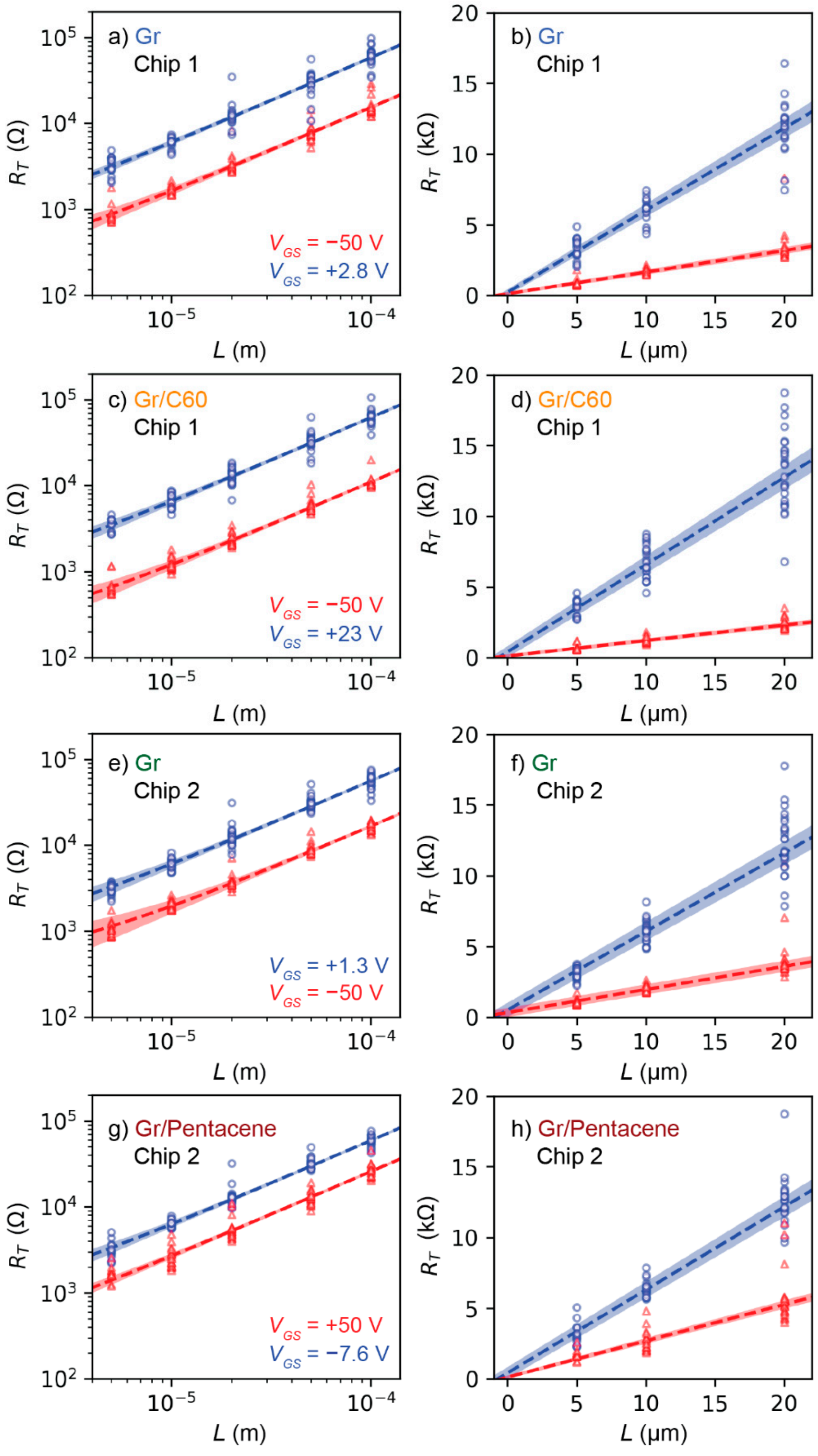

Figure S8. Total resistance (R_T) vs. channel length (L) of the GFETs. The sheet resistance (R_S) and contact resistance (R_C) are obtained from the slope and intercept of the linear regression of the data in the Transfer Length Method (TLM).The red and blue dashed lines are the results of the linear regressions for gate voltages of ±50 V and V_{GS}^{Dirac}, respectively. Figure 4 shows the results of the linear regression for all the gate voltages in the range −50 V to +50 V. (**a**)–(**b**) R_T vs. L of the GFETS before deposition of C60. (**c**)–(**d**) R_T vs. L of the GFETS after deposition of C60.(**e**)–(**f**) R_T vs. L of the GFETS before deposition of Pentacene. (**g**)–(**h**) R_T vs. L of the GFETS after deposition of Pentacene.

Table S1. GFETs measurements population overview. The table shows the number of electrical measurements considered in the analysis. The working device that were considered all show a linear output characteristic (I_{DS} vs. V_{DS}) in the range ±50 mV and the typical graphene field effect transistor transfer characteristic (I_{DS} vs. V_{GS}) in the range ±50 V at a constant V_{DS} = 50 mV.

	**C60-GFETs**		**Pentacene-GFETs**	
**Length (µm)**	**Gr**	**Gr/C60**	**Gr**	**Gr/Pentacene**
5	19	15	18	13
10	16	21	27	21
20	21	25	26	22
50	24	22	26	24
100	21	18	22	18
Working	101	101	119	98
Total	150	150	150	150
